# Diagnosis of Pleural Mesothelioma: Is Everything Solved at the Present Time?

**DOI:** 10.3390/curroncol31090368

**Published:** 2024-08-27

**Authors:** Elisa Roca, Avinash Aujayeb, Philippe Astoul

**Affiliations:** 1 Thoracic Oncology, Lung Unit, P. Pederzoli Hospital, Peschiera Del Garda, VR, Italy; elisaroca@gmail.com; 2 Respiratory Department, Northumbria Health Care NHS Foundation Trust, Care of Gail Hewitt, Newcastle NE23 6NZ, UK; avinash.aujayeb@northumbria-healthcare.nhs.uk; 3 Department of Thoracic Oncology, Pleural Diseases and Interventional Pulmonology, North Hospital, Aix-Marseille University, Chemin des Bourrely, 13005 Marseille, France; 4 La Timone Campus, Aix-Marseille University, 13005 Marseille, France

**Keywords:** mesothelioma, asbestos, thoracentesis, pleuroscopy, pleural imaging

## Abstract

Ranked high in worldwide growing health issues, pleural diseases affect approximately one million people globally per year and are often correlated with a poor prognosis. Among these pleural diseases, malignant pleural mesothelioma (PM), a neoplastic disease mainly due to asbestos exposure, still remains a diagnostic challenge. Timely diagnosis is imperative to define the most suitable therapeutic approach for the patient, but the choice of diagnostic modalities depends on operator experience and local facilities while bearing in mind the yield of each diagnostic procedure. Since the analysis of pleural fluid cytology is not sufficient in differentiating historical features in PM, histopathological and morphological features obtained via tissue biopsies are fundamental. The quality of biopsy samples is crucial and often requires highly qualified expertise. Since adequate tissue biopsy is essential, medical or video-assisted thoracoscopy (MT or VATS) is proposed as the most suitable approach, with the former being a physician-led procedure. Indeed, MT is the diagnostic gold standard for malignant pleural pathologies. Moreover, this medical or surgical approach can allow diagnostic and therapeutic procedures: it provides the possibility of video-assisted biopsies, the drainage of high volumes of pleural fluid and the administration of sterile calibrated talcum powder under visual control in order to achieve pleurodesis, placement of indwelling pleural catheters if required and in a near future potential intrapleural therapy. In this context, dedicated diagnostic pathways remain a crucial need, especially to quickly and properly diagnose PM. Lastly, the interdisciplinary approach and multidisciplinary collaboration should always be implemented in order to direct the patient to the best customised diagnostic and therapeutic pathway. At the present time, the diagnosis of PM remains an unsolved problem despite MDT (multidisciplinary team) meetings, mainly because of the lack of standardised diagnostic work-up. This review aims to provide an overview of diagnostic procedures in order to propose a clear strategy.

## 1. Introduction

Pleural mesothelioma (PM) is a rare, aggressive cancer often linked to previous asbestos exposure [1]. There is usually a lag of approximately 40 years between exposure and disease presentation. Worldwide, the incidence is rising, mostly driven by unchecked asbestos use in countries such as India, Brazil, Russia and China [2,3]. Prognosis is poor, with a median estimated survival of 8–14 months from the time of diagnosis, although widespread use of newer treatments such as immunotherapy might change this over the next few years [4]. Men are more affected than women (up to 80% in some cohorts) [5], with a median age of diagnosis of 74 years. In this narrative review, we present the updated evidence on the pathogenesis of PM, how the disease is commonly present, the various optimal investigative steps, review the histological classifications and updated staging methods, and look to the future for possible new diagnostic techniques.

## 2. Epidemiology and Pathogenesis

Pleura, a serosal surface, is the most frequent localisation of mesothelioma, even if the pericardium, the peritoneum, and the tunica vaginalis can be affected [6]. Pleural mesothelioma (PM) is strongly linked with asbestos exposure, and the disease is more common in men, usually due to occupational exposure. Nevertheless, there is an increasing trend among females, whereas asbestos is less frequently a cause of disease, as recently reported [7,8]. Therefore, despite the ban on asbestos in Western countries, PM still remains a therapeutic challenge despite unequivocal progress, and the most precise understanding of pathogenesis is mandatory. This molecular pathogenesis of PM is multifactorial, driven by asbestos-related and non-asbestos-related mechanisms, which can be summarised as follows [9].

Asbestos fibres are mineral-hydrated silicates divided into two groups: serpentine (curly fibres, ‘white’ asbestos) and amphibole (amosite and crocidolite, needle-like fibres, ‘brown’ and blue’ asbestos, respectively). All types of fibres are carcinogenic, but crocidolite seems to be the most aggressive causative agent, according to the International Agency for Research on Cancer (IARC) [10] but the risk of developing PM is also related to the duration and the heaviness of exposure despite recent results on the animal study [11,12,13,14]. After inhalation and migration to the pleura, fibres lead to a pro-inflammatory environment and induce oxidative and mechanical damage to cells and DNA damage through macrophages and reactive oxygen and nitrogen species (ROS/RNS) [15].

Non-asbestos-related PM can be separated into causative agents, non-asbestos mineral fibres, and non-mineral agents. Erionite and fluoro-edenite, mineral fibres with quasi-similar properties of asbestos, were shown to be carcinogenic in the setting of environmental exposures [16,17]. Regarding the nonmineral sources, radiation has been linked to the development of PM after both therapeutic, in this case usually occurring in irradiated tissue, or occupational exposure [18]. The association between PM and Simian Virus 40 (SV40) in humans is a matter of debate. SV40 is a polyomavirus with oncogenic potential which can induce a mesenchymal cell transformation in vitro and a PM onset in experimental animals. SV40 antibodies and Tag expression in PM patients’ sera samples were found to be significantly higher in comparison to healthy patients, indicating an association. This, however, does not represent proof that SV40 is responsible for the tumour onset [19]. SV40 may function as an exogenous agent that increases the basal level of spontaneous mutations and lowers the threshold for tumour development [20]. Recent studies aiming for an association of PM and SV40 in the Crocidolite-Contaminated area suggested that the occurrence of PM was not related to SV40 infection and that crocidolite exposure was the main cause [21,22].

Besides the environmental exposures, despite a low frequency of protein-altering mutations [23] limiting the potential for molecular targeted therapy, genetic profiling of PM has shown common deletion or loss mutations of genes and germline alterations in CDKN2A, BRCA1, BRCA2, and XPC found to be linked with the development of the disease [24,25]. Among these mutations, somatic or hereditary alterations of the tumour suppressor gene BRCA1-associated protein 1 (BAP1), which produces deubiquitinases enzyme controlling apoptosis, cellular advancement, growth inhibition, chromatin remodelling, and DNA repair response, play an important role in the development of PM [26]. BAP1 depletion is a strong predictive indicator of cancer in mesothelioma differentiation, according to recent studies and predicts improved survival of patients undergoing chemotherapy [9,27,28].

## 3. Presentation

### 3.1. Symptoms

Symptoms differ depending on the type of malignant mesothelioma. Pleural mesothelioma is the most frequent form, and the symptomatology depends mainly on the presence of pleural effusions. In particular, PM patients with pleural effusion may manifest coughing, breathlessness, dyspnoea and chest pain. Chest pain may arise without effusions as well due to chest wall invasion. When the disease is more advanced, there may also be changes resulting from the compression of mediastinal organs, such as the airways, digestive tract or large vessels. As a result, symptoms such as difficulty swallowing, dysphagia, dysphonia, neck and facial oedema may occur.

In addition to this specific symptomatology related to the anatomical changes caused by the pleural effusion, often associated with pathological pleural thickening, the patient may complain of non-pathognomonic and non-specific symptoms such as a general state of malaise, worsening asthenia, muscle weakness and weight loss. As an aside, symptoms of peritoneal mesothelioma are most often non-specific and include weight loss, cachexia, malaise, and asthenia. Instead, the more specific symptoms related to abdominal anatomical changes due to ascites are characterised by abdominal pain, nausea and vomiting, and fever. In these cases, intestinal obstruction, blood clotting abnormalities, anaemia and fever may occur.

### 3.2. Imaging Modalities

The usual first investigative step in PM will be a chest radiograph. Due to the increased pleural surface area of the right hemithorax, asbestos fibres have an increased predilection for the right pleural surface, and right-sided disease is customary (ratio 1.6 to 1) [29]. As explained above, a unilateral pleural effusion is a common finding (pleural malignancies are associated with pleural effusions in up to 94% of cases), and masses can be seen as well [30]. The next accepted step is to have a computed tomography (CT) scan with venous contrast. Peak contrast enhancement in PM occurs after four and a half minutes, so delayed venous phase acquisition of images is required. Leung’s criteria, first described in 1994, have stood the test of time—circumferential pleural thickening, nodular pleural thickening, parietal pleural thickening greater than 1 centimetre, and mediastinal pleural involvement have specificities of 94%, 94%, 88%, and 100% respectively, and sensitivities of 51%, 36%, 56%, and 41% [31]. However, approximately 40% of CT scans can be reported as benign despite an underlying malignant diagnosis, and almost 50% of patients with PM can have a benign CT report without specialist thoracic radiology reporting [32]. This is even lower with CT pulmonary angiography (27%). With specialist reporting, reported sensitivity and specificity can be much higher [33].

At the time of pleural fluid intervention (which is discussed later), ultrasound imaging is currently mandated. The sonographic features of PM are similar to any malignant disease- nodular pleural thickening of more than 1 centimetre and diaphragmatic nodularity have high specificity (95–100%) but lack sensitivity (40%) [34].

Other imaging modalities have been studied. Positron Emission Tomography (PET)-CT can help evaluate distant and nodal disease, but PM has relatively low metabolic activity. Thus, patients with early-stage disease could have a false negative PET scan and patients with previous pleurodesis or simultaneous inflammatory conditions such as rheumatoid arthritis can have false positive scans [35,36]. A previous meta-analysis concluded that PET CT should not be recommended for distinguishing between malignant and benign effusions [37]. The value of PET CT in obtaining biopsies will be discussed later. Magnetic resonance imaging (MRI) has shown promise in clinical trials. MRI is very good for soft tissue characterisation and is better than CT for assessing chest wall and diaphragmatic invasion. However, its use is not widespread due to associated costs and service provision implications. The sensitivity of MRI can be as high as 92% in selected patients [38].

## 4. Diagnosis

### 4.1. Diagnostic Evaluation—Pleural Effusion Investigation and Tissue Biopsies

As explained above, the vast majority of pleural malignancies present with a pleural effusion. Under thoracic ultrasound, the pleural effusion can be identified, and a sample can be taken (a pleural tap) if it is safe to do so. Up to a litre of fluid can be removed at the same time for relief of breathlessness if required (therapeutic aspiration). Pleural fluid analysis should then ensue to determine if it is an exudate or a transudate according to Light’s criteria (pleural fluid is considered an exudate if pleural fluid protein/serum fluid protein ratio > 0.5, pleural fluid lactate dehydrogenase (LDH)/serum fluid LDH ratio > 0.6, or pleural fluid LDH > 2/3 of the upper limit of normal serum LDH). Malignancies are often associated with exudative effusions, although up to 10% of transudative effusions can be malignant. Alongside biochemical analysis, cytological assessment of the fluid is important. As with any test, the pre-test probability is important. Previous research has shown that cytology is helpful in less than 6% of cases of PM, but sensitivity can be as high as 95% in patients with ovarian or breast cancer [39]. As such, a direct biopsy approach in patients with a high clinical suspicion of mesothelioma is advocated by many centres, and this has been suggested in the updated British Thoracic Society 2023 Pleural disease guidelines [40]. Pleural fluid cytology cannot also determine the extent of tumour invasion, although it is in favour of visceral pleural involvement [41,42].

There are three ways to obtain a biopsy: ultrasound-guided, CT-guided, or local anaesthetic thoracoscopy (LAT). For actionable molecular profiling, tissue is required in the form of a pleural biopsy. Sundaralingam et al. have shown that the highest yield for successful molecular marker analysis was from LAT procedures (95%). CT and ultrasound-guided biopsies had 86% and 77% yield, respectively (*p* = 0.004) [43]. LAT is the preferred option for PM diagnosis, with diagnostic yields that are often quoted as above 95% and very low complication rates. It offers a therapeutic (all the associated pleural fluid can be drained for symptom relief), diagnostic (areas of pleural malignancy can be biopsied under direct vision) and preventative (talc pleurodesis via poudrage with or without insertion of an indwelling pleural catheter). The various techniques regarding the LAT procedure are beyond the scope of this article but are well described elsewhere [44]. However, patients have to be adequately fit to undergo LAT, and if LAT is not feasible and an obvious radiological target is present, such as easily visible parietal nodules, image-guided biopsies can be performed. Whilst CT-guided biopsies are the exclusive remit of radiologists, ultrasound biopsies are increasingly being performed by respiratory physicians with good, reported outcomes [45,46].

PET-CT has been used previously to aim for pleural tissue that shows up metabolically active despite the aforementioned limitations [47]. The recent TARGET trial showed that PET-CT is not useful in guiding new pleural biopsies in those patients who have undergone a previous non-diagnostic biopsy, so it seems that PET does not have much of a role to play in mesothelioma diagnostics [48]. It is only recommended in patients to elucidate signs of distant disease [30].

### 4.2. Molecular and Genetic Markers

The molecular landscape of PM is characterised by heterogeneity in the inactivation of tumour suppressors and the activation of specific targets that could represent a target for new personalised therapies.

Potential molecular targets for PM could be represented by alterations involving genes that play a role in cell cycle regulation. Out of them, the homozygous deletion of 9p21 can be detected in MPM in 50–75% of cases [49], and this genetic alteration can involve Cyclin-Dependent Kinase Inhibitor 2A (CDKN2A) and methylthioadenosine phosphorylase (MTAP). Other molecular markers are represented by gene coding for receptor tyrosine kinases. Out of them, epidermal growth factor receptor (EGFR), which is known to be involved in the proliferation and regulation of cell growth as well as in the angiogenesis process, is often overexpressed in PM (about 40–90%) [50,51]. For this reason, many studies have been aimed at the application of EGFR inhibitors in PM but failed to demonstrate significant clinical benefit [52,53,54,55]. The reasons for the lack of effectiveness of these drugs are probably manifold. Indeed, despite the overexpression, EGFR mutations or amplification are very uncommon in PM. Moreover, there may be concomitant genetic and molecular alterations that activate resistance mechanisms [56]. Another family of receptor tyrosine kinases, usually expressed in solid tumours and among them in PM, is represented by the TAM receptors (Tyro3, Axl, and Mer) [57]. The TAM family proteins are demonstrated to play an important role in tumour development and progression, metastasis, and microenvironment alteration, often resulting in drug resistance [58,59]. Other genetic alterations could represent a target for PM, such as modifications in genes involved in the Hippo Signaling Pathway [60,61,62]. In particular, the neurofibromatosis type 2 (NF2) tumour suppressor gene is frequently detected in PM as somatically mutated [63]. In PM, it is possible to find alterations in this gene in about 50%, such as non-sense or missense mutations, gene rearrangements, and deletions with a loss of heterozygosity resulting in bi-allelic loss of function [64].

NF2 gene encodes Merlin protein, which plays a crucial role in cell proliferation and survival, cellular signalling pathways, and balancing oncosuppressors and oncogenes [65,66,67,68]. The Hippo pathway components have tumour suppressive activity and are represented by LATS1/2 (large tumour suppressor kinase 1/2), MST1/2 (mammalian STE20-like protein kinase, (SAV1) Salvador homolog 1, and (MOB1) kinase activator 1A/BPM has been shown to be related to Hippo pathway dysregulation, involving YAP/TAZ oncoproteins and LATS1/2 tumour suppressors, through the activation of specific mechanisms: tumour initiation, progression, metastasis, and drug resistance [60]. YAP and TAZ activation results in regulating genes useful for transcription, such as TEAD1–4 [61,62].

In close association with the Hippo-dependent processes, the PI3K pathway is often activated in MPM, and it has been shown to be involved in tumour cell survival and proliferation [69]. PM can also be characterised by molecular and genetic alterations involving enzymes aimed at cellular metabolism. Out of them, ASS1 (argininosuccinate synthetase1) is an enzyme precursor for several molecules involved in tumorigenesis, and it is often (in about 45–65%) downregulated in non-epithelioid PM [70,71]. Another enzyme involved in metabolism is glutamine, a substrate used in redox homeostasis, Krebs cycle, and the synthesis of nucleic acids. YAP1/TEAD pathway influences glutamine signalling [72]. Out of surface targets, mesothelin is one of the most studied cancer-associated antigens overexpressed on the membrane of PM cells, which seems to have a role in tumour development, metastasis and drug resistance [73,74,75,76,77,78,79,80,81,82]. The soluble form of mesothelin results from the cell membrane release promoted by proteases [83]. Healthy pleural, pericardium and peritoneum mesothelial cells poorly express mesothelin; this underlines how this molecule could be considered an ideal biomarker to design target therapy [84,85]. Regarding possible surface targets for patients affected by PM, another ideal antigen for targeted treatments is the oncofoetal glycoprotein 5T4, given its high expression on mesothelioma cell lines [86]. PM can also be characterised by germinal and acquired mutations in genes involved in response to DNA damage. Indeed, genes involved in DNA repair pathways are frequently found in PM. Out of these genes, BRCA1-BAP1 (breast cancer gene 1-associated protein 1) is the most common in PM (approximately 60%) [81,87,88]. EZH2 (enhancer zeste homolog 2) is an enzyme oncogenic driver regulating gene expression carcinogenesis and is required for lung mesothelium differentiation [89,90]. Moreover, several studies suggest that an impaired DNA repair system influences PM pathogenesis by leaving uncorrected genomic alterations [91]. Given the role of BRCA1 in PM and the involvement of BAP1 and BRCA1 in the DNA damage response, these genetic alterations could be targeted as biomarkers, using, for example, PARP inhibitors (PARPi) [92].

The exhaustive characterisation of the phenotypes of PM and the pathogenetic mechanisms that determine its development and evolution remain unclear. Recent multi-omics analyses aim at the detection of ideal markers based on genetic and molecular alterations. The integration of anatomopathological analysis in association with the definition of biomolecular and genetic characteristics may provide a more precise picture of the disease and novel therapeutic approaches [93].

### 4.3. Potential New Tests for Diagnosis (Breath Test…)

A number of markers for PM have been studied (or are being studied in large-scale mesothelioma trials such as ASSESS-Meso and Meso-Origins). First of all, there are pleural and serum mesothelin (or Soluble Mesothelin Related Peptides [SMRP]) levels. Previous studies have shown that more than 80% of PM cells can express mesothelin, but overall sensitivity and specificity of serum mesothelin levels are at 0.61 and 0.8, respectively [94]. SMRPs were studied in the SWAMP study, and a fall in SMRP between baseline and 8 weeks after chemotherapy would suggest stability of disease burden, at least on contemporaneous imaging. Lower SMRP levels at completion of treatment are also associated with better survival [95]. Further prospective studies looking at SMRPs, such as a sub-study of ASSESS-meso, are currently finished and will be reported soon. Pleural fluid mesothelin has also been studied, as mesothelin is secreted directly from the mesothelial cells into the pleural fluid [96,97]. Pleural mesothelin levels are increased, as Pass et al. demonstrated, but so far, they have not been proven to be a reliable marker of disease. Other markers such as Fibulin-3, Osteopontin, Megakaryocyte potentiating factor (MPF and Hyaluronic acid (HA) have all been studied but never prospectively, and none are recommended for routine use [98].

There has been an interest in volatile organic compounds (VoCs) from exhaled breath for many years now. VoCs have previously been shown to discriminate between patients with PM and patients with high asbestos exposure, as well as patients with benign asbestos lung disease [99]. More recent refinements of the process have suggested that some VoCs could have 100% sensitivity and specificity, but only 7 PM patients were studied [100]. Large-scale validation of these breath tests in areas of high and low prevalence is required.

### 4.4. Liquid Biopsy in PM

In the oncologic landscape, liquid biopsy is increasingly applied for early identification of at-risk subjects, diagnosis, treatment monitoring, disease progression and prognosis. However, the innovations achieved in this field have not been translated into the clinical practice of PM patients and promising non-invasive markers [101,102,103]. In this context, several markers have been analysed, and research efforts have been made to identify an ideal, non-invasive, and effective biomarker to follow patients with MP.

Among these, several studies have focused on their possible applicability in clinical practice: proteins such as mesothelin [104,105,106,107,108,109,110], osteopontin [105,111,112], Fibuline-3 (FBLN3) [109,110,113], High-mobility group box 1 (HMGB1) [114,115,116], CD138 [117], angiogenic factors [118,119], microRNAs [120,121], circulating tumour DNA (ctDNA) [122,123,124], circulating tumour cells (CTCs) [125,126], exosomes [127].

However, although there are many candidate biomarkers, only mesothelin has received Federal Drug Agency approval, although it has a low diagnostic sensitivity. OPN is a marker for the duration of asbestos exposure and has a potential prognostic role, but it lacks specificity for PM.

Proteomic approaches have also been applied to define predictive prognostic signatures, but these results are still in the research phase [128,129,130]. Analysis of epigenetic features could also lead to innovative approaches for PM pathology. However, these findings require further validation and confirmation on large samples [131].

To date, despite interesting perspectives, the use of circulating biomarkers and liquid biopsies in current practice for the management of pleural mesotheliomas is not clearly defined [132,133].

### 4.5. Artificial Intelligence for PM

Artificial intelligence (AI) has been researched in the diagnosis of various disease conditions. Indeed, the application of AI for PM patients could have great potential in facilitating the diagnosis [134]. In this context, researchers have analysed clinical, radiological, and biological variables for PM patients to propose innovative diagnostic methods using artificial intelligence techniques.

Latif et al. used databases of PM patients to extract PM-related symptoms to identify the risk factors for this neoplasm as early as possible. The authors of this research believe that artificial intelligence and data analysis of PM patients could be useful not only in early diagnosis but also in the management of comorbidities of patients with this disease [135]. Other research has been developed to define the best possible system for the early identification of individuals at risk of developing PM and patients with a worse prognosis. In this field, AI-based algorithms were applied to develop experimental models defining specific risk and prognostic factors for this disease [136]. Likewise, some scientists have deepened the study of PM risk variables by including the characteristics of both patients and healthy subjects in the analyses in order to have larger databases [137].

Several machine-learning algorithms were also applied to detect PM patients at an early stage. The techniques used in this research were resampling, adaptive synthetic sampling (ADASYN) and minority synthetic oversampling technique (SMOTE) [138].

A useful method for predicting the survival of PM patients from specific images was also devised: the MesoNet [139].

The ability to identify individuals at risk of developing cancer at an early stage is one of the most important frontiers in medicine. Therefore, artificial technologies can contribute to the development of AI models for early diagnosis, treatment monitoring, and definition of prognosis.

In particular, AI could offer a rapid, effective and non-invasive method for diagnosing patients with PM. However, artificial technology is not yet applicable in clinical practice due to certain limitations and shortcomings, as well as the complexity of the healthcare business. Optimising learning processes and improving data classification will lead to improvements in the field of AI applied to medicine, complementary to current diagnostic methods.

## 5. Staging and Histologic Classification

### 5.1. Staging

The 8th TNM revision, carried out by the mesothelioma staging project experts from the IASLC (The International Association for the Study of Lung Cancer), was obtained through the analysis of huge amounts of data from MPM patients (>3500) [140,141,142]. The stage of the disease is of paramount importance in defining the most appropriate course of treatment for the patient. In particular, it can point towards therapeutic interventions aimed at prolonging survival and improving outcomes rather than palliative therapies alone.

Among non-invasive staging techniques, a CT scan is the first approach for both the definition of active anticlastic treatment for patients who can benefit from it and for unfit patients who will be referred to palliative care. Indeed, in these cases, a CT scan can be useful during the planning of a palliative thoracoscopy with eventual talc pleurodesis [141].

PET-CT can be used to perform lymph node staging and to detect rare distant metastasis, although the results of this diagnostic technique can often be controversial with the presence of false positives [143,144,145].

MRI is usually not performed for PM, except for the analysis of the most peripheral areas (the apices, the subclavicular vessels, the diaphragmatic areas…), which are useful for defining the resectability of the disease [146]. Although the rate of metastasis of PM is very rare, MRI can be carried out to identify brain metastases more sensitively than CT; however, it is not superior in detecting lymph node metastases or visceral pleura tumours [147]. In clinical practice, the application of MRI remains limited, as it is preferred to use CT scan or PET-CT; MRI-based staging approaches are currently only applied for research purposes [148]. Among invasive techniques, mediastinoscopy can be applied as a procedure to analyse the mediastinal lymph nodes [149,150].

Bronchoscopy with EBUS is another technique routinely used for lymph node staging of thoracic tumours and is sometimes also applied for PM [151,152].

EUS is very rarely used to study suspicious lymph nodes on radiological evaluation in patients with PM [153]. Out of other invasive staging techniques, thoracoscopy and laparoscopy can be applied, although this happens infrequently and only to identify stage IV patients not diagnosed by PET-CT [154].

### 5.2. Histologic Classification

Adequate tissue specimens are needed for PM diagnosis, which remains purely histological, based on specific and validated pathological classifications defined by experts throughout the world [155,156,157,158]. Pleural effusion is one of the most common presentations of PM. Therefore, cytology is the first diagnostic technique to be carried out. In these cases, cytological procedures allow us to distinguish between benign and malignant pleuritis [159]. However, even after obtaining the cytological diagnosis, tissue confirmation remains crucial. Indeed, the sensitivity of cytological diagnosis is about 30–75%, similar to that achieved by fine-needle biopsies, and certainly lower than with pleural biopsies [160,161]. Nevertheless, whether the patient is not amenable to biopsy (poor performance status, comorbidities, concomitant medications…), the diagnosis can be ascertained on cytology alone [39,157].

More often than not, a definitive diagnosis derived from biopsy material in the correct quantity and quality is needed in order to allow conclusive characterisation [162]. Moreover, the specimen quality may affect the accuracy of histological classification and subtyping.

Macroscopic analyses are fundamental in the PM diagnostic process, considering that the topographical features of the tumour are crucial for pathological staging, as well as the fact that mesothelioma varies during the tumour’s natural history.

Differentiating between the different types of PM and pleural metastases from other primary neoplasms (lung, breast, etc…) is achieved through the application of immunohistochemical analysis and specific sets of antibodies. In addition to this, claudin 4 has recently been studied, which would appear to be very useful in the differential diagnosis between PM and adenocarcinoma [157]. Cytokeratin remains a very useful marker for defining sarcomatoid MPM [159].

PM can be classified into three major histological subtypes: epithelioid, sarcomatoid, and biphasic. It is also used as a prognostic and predictive factor for specific therapy. However, exhaustive implementations of this codification were introduced thanks to the 2021 WHO Classification of Tumors of the Pleura [163,164]. In particular, several studies investigated the importance of different cytologic characteristics, architectural patterns, and stromal features as prognostic factors useful in identifying patients candidates for multimodal treatment [165].

Another factor associated with prognosis is grading; indeed, specific morphological features, such as mitotic count, nuclear atypia, and necrosis, could be used for risk stratification and the definition of more personalised therapies [166,167,168].

Current classification systems for MPM have, therefore, been updated to include specific features such as architectural pattern definition, stromal and cytologic characteristics, and biological and molecular features in the pathological analysis [164].

The 2021 WHO Classification of Tumors of the Pleura offers important changes compared to past classifications. More in detail, these are the most crucial changes: the renaming of WDPM (well-differentiated papillary mesothelioma) in WDPMT (well-differentiated papillary mesothelial tumour) [169], the recognition of mesothelioma in situ as a defined pathologic entity [161,170,171,172], the incorporation into the 2021 classification of the architectural, cytologic and stromal features of the three well-known histological classifications (epithelioid, sarcomatoid and biphasic) because of their prognostic role, and the introduction of nuclear grading for epithelioid diffuse mesothelioma.

## 6. Conclusions

The above narrative review gives an overview of the diagnostic pathway of pleural mesothelioma. The main limitation of this review is that it effectively constitutes an expert review of the author’s opinions and practice. However, we believe that this will be informative and practical for the general readership. Future work should concentrate on the widespread application of ancillary molecular diagnostic tests (there is great inequity in those), on rigorous appropriate staging and clinical use of MRI, on the effective use of non-endoscopic pleural biopsies and the appropriate use of AI without replacing the human touch.
